# Correlates of psychological distress and self-rated health among Palestinian citizens of Israel: findings from the health and environment survey (HESPI)

**DOI:** 10.1186/s13584-021-00439-z

**Published:** 2021-01-20

**Authors:** Mohammad Khatib, Ivonne Mansbach-Kleinfeld, Sarah Abu-Kaf, Anneke Ifrah, Ahmad Sheikh-Muhammad

**Affiliations:** 1 The Galilee Society, the Arab National Society for Health Research & Services, PO, Box 330, 20190200 Shefa-‘Amr, Israel; 2 Department of multidisciplinary studies, Ben-Gurion University of the Negev, Be’e Sheva, Israel; 3 Israel Center for Disease Control, Ministry of Health, Ramat Gan, Israel

**Keywords:** Psychological distress, Self-rated health, Palestinian-citizens-of-Israel, GHQ

## Abstract

**Objectives:**

Psychological distress is a problem strongly associated with socio-economic conditions. This study aims to assess rates of psychological distress and ‘poor’ self-rated health among Palestinian citizens of Israel, who constitute 21% of the population and nearly 50% live in poverty, and compare their psychological distress scores with those of the general Israeli population.

**Design:**

The Health and Environment Survey among Palestinian citizens of Israel (HESPI-2015), included a representative sample of this minority; 2018 individuals aged ≥18 were interviewed. The questionnaire included socio-demographic and health-related information, the General Health Questionnaire-12, (GHQ-12) and Self-rating of health (SRH).

**Results:**

Subjects with GHQ-12 global scores of ≥17 were considered to have high distress. Low education, female gender, obesity and the presence of chronic diseases were significantly associated with high psychological distress over and above the effect of the other variables. Poor SRH was strongly associated with having a chronic disease and additional risk factors were older age group, low education and high psychological distress. Comparing psychological distress scores of Arabs in Israel with those of the general population showed that 30% of the former were classified as asymptomatic, as compared to 75% in the general population while the proportion of symptomatic or highly symptomatic was 14% in the latter as compared with 45% in the former.

**Conclusions:**

It appears that the burden of poverty, chronic disease and low education in this population, which suffers from multiple stressors, is disproportionate and should be addressed by the authorities, together with concrete plans to improve the education of the younger generations. Clearly, the association between discriminating policies and deprivation with psychological distress is not unique to the case of the Palestinian minority in Israel and therefore this study will allow for the examination and generalization of the current findings to other discriminated and disadvantaged minorities.

**Supplementary Information:**

The online version contains supplementary material available at 10.1186/s13584-021-00439-z.

## Introduction

Psychological distress is recognized worldwide as a public health problem that affects quality of life [1, 2]. Psychological distress has been defined as consisting of symptoms of anxiety and depression, social dysfunction and inability to cope with daily activities [3, 4]. Most epidemiological studies have found that it is strongly associated with adverse socioeconomic conditions [5–7], to the extent that it has been considered “an efficient indicator of the psychological impact of adverse conditions present among low status community groups” [[8], p. 64]. The link between psychological distress and mental disorders, assessed with the DSM-IV or ICD-10, has also been empirically established [9, 10]; although among minority groups the assessment of this link has shown inconsistent results. Some studies that showed high distress scores among disadvantaged minority groups have found that these high distress scores are not “significantly associated with differential rates of common mental disorders” [[11], p. 111]. This same inconsistent relation between psychological distress and mental disorders is shown by Barnes & Bates (2017), who found lower prevalence of major depressive disorders among Blacks relative to Whites, despite greater socio-economic deprivation and worse physical outcomes among the former [12]. One explanation for this paradox is that psychiatric questionnaires may not be tapping mental disorders accurately among marginalized groups compared to non-marginalized groups [13]. Another explanation refers to the possibility that screening instruments in community-based studies might address symptoms that reflect experiences of psychological suffering more accurately than those presented in psychiatric questionnaires tapping mental disorders [10].

### Psychological distress, demographic, socio-economic, health-related and community factors

Studies have found that high psychological distress is consistently associated with female gender [14–16], and low educational level [9, 17, 18]. Regarding age, findings are not always consistent. Some studies have found a decline in psychological distress with increasing age, offering the explanation that a sense of coherence, self-esteem and feelings of happiness increase with age and peak between 40 and 59 years of age [19, 20]. Studies among young African American men have found higher rates of psychological distress than those over 35 years of age due to their exposure to more frequent and severe stressors [12]. In other population groups, however, studies have found that distress scores increase with the health problems and loneliness associated with age [11, 18].

The increased psychological distress found among those in the very-low income groups may be attributed to the possibility that factors associated with poverty have become “more powerful risks for emotional difficulties over time … and might lead to a disproportionate increase in emotional problems in low-income groups” [[21], p. 1086], and that the gap between those in the very low socio-economic levels and those slightly above them, with respect to exposure to adverse life events, maternal distress and family dysfunction, has increased [22]. Underprivileged groups are also affected by more negative life events, by chronic stress that cannot be eased promptly and by social networks that are not always reliable as compared to those of higher income groups [9, 23].

Other health-related risk factors for psychological distress and depression include obesity and chronic disease [24], and community factors such as neighborhood characteristics [25].

Veenstra (2011) suggests that we perceive the inequalities in health that are associated with gender, education and socio-economic position as intrinsically entwined and analytically inseparable as they mutually reinforce one another. “Disadvantaged identities experienced in tandem are seen to result in inordinate, i.e., even more than additive, amounts of disadvantage … rather than a simple cumulative or mitigating effect” [[26], p.3].

The Israel National Health Survey conducted in 2003–2004, measured psychological distress using the General Health Questionnaire-12 (GHQ-12) and found higher prevalence of highly symptomatic psychological distress among women than among men (7.7% vs 5.1%, respectively), among those with low education than among those with an academic education (18.4% vs. 2.3%, respectively), among the obese than among those with normal/low weight (10.5% vs. 4.2%, respectively), and among those with a mental disorder as compared to those without a mental disorder (17.1% vs. 4.0%, respectively) [18].

### Palestinian citizens of Israel and risk factors for psychological distress

Palestinian citizens constituted 21% of the total Israeli population in 2018 [27]. Over 85.0% were Muslim, 7.3% Christian, and 7.7% Druze [27, 28]. Data published by the National Insurance Institute [29], reported that 45.0% of Arab families in Israel, 57.8% of their children and 55.9% of their elderly were found below the poverty line, while the average poverty rate for the general Israeli population in 2018 was 18.0%, and for children it was 30.0%. In 2011, after tax and transfer payments, rates of Palestinian citizens below the poverty line were reduced from 58 to 51%, whereas among the Jewish population they were reduced from 28 to 15% [30]. In addition to the low income and levels of poverty, there is a lack of development and government investment in infrastructure, education and health services in the Arab cities and towns [31]. This is particularly widespread in the Southern District, where 17% of the Arab citizens of Israel live [25]. The Southern District includes the Bedouin population, the poorest Israeli citizens, with a high birth rate. About 37% of them live in 45 ‘unrecognized villages’ whose existence is not officially recognized by the State of Israel. These unrecognized Bedouin villages are characterized by a lack of governmental provision of basic services, such as sewage, running water, educational institutions, primary care clinics, mental health facilities and public transportation, among others. These bleak conditions strongly impact the socio-economic status of the families, of whom 64.2% live in poverty [32].

This institutional discrimination of Palestinian citizens of Israel has been legally endorsed with the adoption of the Nation State Bill (Basic Law: Israel: The Nation-State of the Jewish People), on 19.7.2018 by the Israeli Parliament, causing additional anxiety and concern in the Arab population.

The Palestinians in Israel also suffer from an excess of chronic diseases and unhealthy behaviors known to be associated with poverty and marginalization. The Israeli National Health Interview Survey (INHIS) of 2014–2015 [33], found significantly higher age-adjusted prevalence of multiple chronic conditions (MCC) among the Arab population than among the Jewish population.

The conditions of discrimination and social marginalization experienced by the Palestinian citizens of Israel [28, 34–36], are reflected in their higher levels of psychological distress when compared with the Jewish majority [11, 37]. A study among Israeli citizens over 60 years of age found that 33% of Arab men and 45.7% of Arab women reported high psychological distress, as compared with 16.5% of Jewish men and 25.2% of Jewish women [8].

### Self-rated health (SRH)

SRH is a single-item ordinal measure frequently employed as an indicator of general health status or well-being in epidemiological studies as it has high predictive and concurrent validity [38], and captures elements of health such as vitality, that others measures do not tap [39]. SHR has shown predictive power for later morbidity, mortality, the use of health services and disability pensioning [40]. Studies among ethnic minorities have found associations between poorer SRH and female gender, older age, depressive affect, low socioeconomic status, cognitive impairment, low self-esteem, discrimination, chronic disease, co-morbidity and neighborhood characteristics, while factors positively associated with better SRH have been identified as higher education, better physical and mental health and social support [41–43]. Bombak and Bruce (2012) claim that … [In] “societies in which socio-economic disparities are especially pervasive and disadvantage [is] visible [among] minorities and indigenous populations, somatic, psychological, and subsequent self-rated health may suffer” [[44], p. 6]; this may partly be explained by the stress induced by a lack of education and employment opportunities, discrimination, and acculturation and language tensions.

Zajacova, Huzurbazar & Todd (2017), examined the interaction between age and gender and found that both “mid–life and older men weigh physical functioning deficits and negative health behaviors more heavily than women” [[45], p. 58], and that although younger women report worse SRH than men, this trend is reversed at older ages.

Two methodological questions require our attention. The first is to what extent cultural factors affect perceptions of health, of conceptualization of what constitutes health and of which factors are important in the self-assessment of health among different ethnic groups; since cultural variations may produce different findings regarding the weight of specific factors in diverse populations [44]. The second is how these self-evaluations are reached. According to Bailis, Segall & Chipperfield (2003), one possible interpretation is that they reflect a spontaneous assessment of one’s health status and another, that they reflect an aspect of one’s enduring self-concept. If self-evaluation measures health status then it should change whenever there is change in other variables that are closely associated with health status. However, if it relates to the individual’s self-concept of health then it should show stability independently of the observed health changes during this period [46].

This paper uses data obtained by the Health and Environment Survey of Palestinian citizens of Israel (HESPI), and presents the findings with regard to rates of psychological distress, measured with GHQ-12, and of ‘poor’ self-rated health, assessed with the Self-Rated Health (SRH) ordinal measure. It also presents the socio-demographic and health-related risk factors associated with each of these widely used measures. Our objectives were: 1) to assess the rates of psychological distress and ‘poor’ SRH among Palestinian citizens of Israel according to selected socio-economic and health- related factors, and 2) to compare the distribution of psychological distress scores in our sample with that of the general Israeli population and to offer possible explanations for the differences.

## Materials and methods

This article is based on part of the extensive data collected by the Health and Environment Survey among Palestinian citizens of Israel (HESPI), carried out by Rikaz – Applied Social Research Center of The Galilee Society, between November 2015 and February 2016. This cross-sectional survey included a representative sample of Palestinians in Israel (*N* = 2246 households). Nine thousand sity-three individuals were interviewed and 2018 aged 18 or older, participated in a follow-up in-depth interview (971 men and 1047 women).

### The target population

The target population included all Arab Palestinian households in Israel in 2015.

*The sampling frame* included segregated Arab cities and towns and mixed Arab-Jewish cities. The categories published by the Israeli Central Bureau of Statistics (CBS) in the 2008 Population Census were used to determine the enumeration areas [47]. These areas were used as preliminary sampling units (PSU) in the initial selection stage.

#### Sampling design

The sample was designed as a three-stage stratified cluster with systematic random sampling. In the first stage, a systematic random sample.

comprising 75 districts was chosen. In the second stage a sample of 30 households in the selected enumeration district was drawn, and in the third stage an individual aged 18 years or older from each household was selected, using Kish spreadsheets for random selection [48, 49]. The selected individuals participated in the follow-up in-depth interviews. The study population was stratified by gender and age.

#### Calculating sample size

Sample size was determined so that it would provide adequate statistical power for comparisons of subjects with and without one or more chronic conditions. The prevalence of one or more chronic conditions, according to previous data, was 14.5% [50]. The necessary sample size was estimated to include 2250 families.

### Procedures

#### Mechanism for sampling households

A systematic sampling method was utilized for reaching households in each enumeration area until 30 households were reached. A household member aged 18 or older was selected to be interviewed. If the selected member was absent, the interviewer visited the household one or two additional times to complete the questionnaire. Quality control procedures were carried out by the field coordinator who reviewed the completed questionnaires, studied the reports and comments and reviewed each interviewer’s filled questionnaires.

#### Response rates

Out of the 2246 sampled households, 2018 participated in the study, providing a response rate of 89.8.

Instruments:

- The socio-demographic questionnaire was prepared ad hoc and included, among other variables, gender, age, educational level, geographical district and income. Income was used to calculate whether the family was below or above the poverty line. The family was considered to be below the poverty line if the total net income was less than 2526 NIS as of 2015 [51].
- The health-related questionnaire was also prepared ad hoc and included, among other variables, the presence of chronic diseases and weight and height.
- The General Health Questionnaire-12, (GHQ-12), developed to assess the presence of psychological symptoms of distress [52], was used. Subjects were presented with 12 symptoms and were asked whether they experienced a particular symptom or behavior in the past 4 weeks. Response categories were: 0 = never; 1 = rarely; 2 = sometimes; 3 = often. Thus, the range of individual item scores was between 0 and 3 and of global scores between 0 and 36. This rating method has been considered the most appropriate for statistical purposes [53]. A review of several studies [54], indicated that the most common cut-off score for high distress was 2/3, or the 67th percentile. In our study, all subjects in the highest 1/3 of the score distribution were classified as having high psychological distress and those in the lower 2/3 of the score distribution were classified as having low psychological distress. The cut-off score was 17; thus, subjects with a global score of 17 or more were included in the high psychological distress category. In the current study, we used the Arabic version of the GHQ-12 [55]. Cronbach alpha coefficient of the GHQ-12 for this population was 0.885 (0.887 for men and 0.878 for women).
- Self-rating of health (SRH) was assessed by means of the following question: “How would you assess your health?” The respondents were given 4 options: ‘excellent’, ‘good’, ‘not good so good, ‘poor’. This single item ordinal measure has been found to have high predictive and concurrent validity [38–40]. SRH and the GHQ-12 have been found to be very sensitive for identifying distress and well-being in community studies [56].

### Data analysis

Analyses were performed using the SPSS-25 Software package (SPSS Inc., Chicago, IL, USA). Chi square for the association between high psychological distress and self-rated health and individual variables were calculated. The significance level was set to equal to or below 0.05 and was based on the Mann-Whitney test (two independent samples). Logistic regression analysis was performed to test for the association between the variables found in the univariate analyses to be significantly associated with high psychological distress and poor self-rated health. Internal consistency of the GHQ-12 and distinct scales was assessed by means of Cronbach’s alpha.

**Fig. 1:**
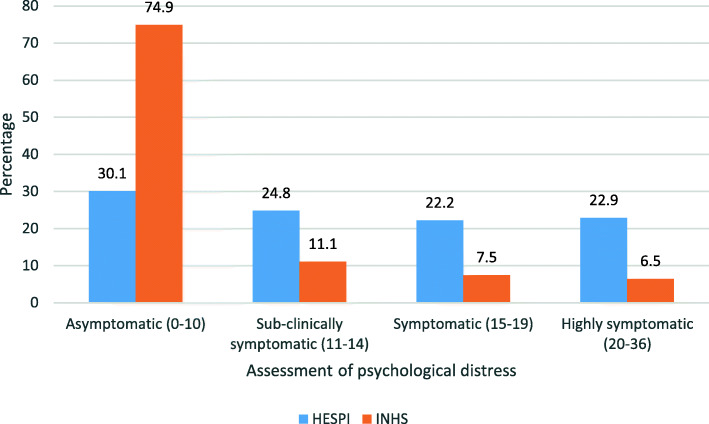
Distribution of psychological distress in the HESPI and INHS

In order to be able to compare our findings regarding psychological distress with those of other Israeli studies, it was necessary to re-categorize our data. The studies we chose for comparison analyzed GHQ-12 in different ways - some using categories with different cut-off points, others dichotomizing the data and still others using mean total scores. In Fig. 1 our data are presented using the four categories proposed by Ponizovsky et al. (2018) [18].

### Findings

#### Characteristics of the population

Table 1, in the left side columns, shows the distribution of selected characteristics of the population by gender. 15.1% of the sample were between 18 and 24 years of age and 10.7% were aged 65 or above. No gender differences in age were found. Educational level differed significantly by gender: more women than men had no education at all (*p* = .011), while more men than women had only partial high school studies (*p* = <.001). There were no gender differences regarding achievement of a high school diploma or an academic education (12.5% of the study population). Educational level also differed by age, since 19.5% of those aged 25–44 had an academic education compared to 1.4% of those aged 65 or more and only 1.4% of those aged 25–44 had no education as compared to 59.5% of those aged 65 or more (χ^2^ = 1042.6; df = 16; *p* = < .001) (data not shown). A striking trait of this population is the high level of poverty: 42.7% of the study population were defined as being below the poverty line; no gender differences were found. It must be stressed here that poverty data, based on income information, is missing for 14.1% of the subjects (127 men and 158 women).

**Table 1:** Rates of high psychological distress among Palestinian citizens of Israel by socio-demographic and health-related factors

Socio-demographic and health-related variables	Distribution of population’s characteristics by gender (%)			Rates of high psychological distress (mean global GHQ-12 score = ≥17), by gender			
	Male	Female	Total	Male	Female	Total	*P* value for gender differences within each sub-category
	N (%) 971 (48.1)	N (%) 1047 (51.9)	N % 2018 (100)	N (%) 277 (28.5)	N (%) 430 (41.1)	N (%) 707 (35.0)	
				χ2 = 34.8; df = 1; *p* = < 0.001			
**Age**							
18–24	137 (14.1)	167 (16.0)	304 (15.1)	34 (24.8)	50 (29.9)	84 (27.6)	.320
25–34	237 (24.4)	285 (27.2)	522 (25.9)	64 (27.0)	90 (31.6)	154 (29.5)	.254
35–44	249 (25.6)	228 (21.8)	477 (23.6)	55 (22.1)	82 (36.0)	137 (28.7)	.001
45–64	250 (25.7)	249 (23.8)	499 (24.7)	73 (29.2)	119 (47.8)	192 (38.5)	.000
65≥	98 (10.1)	118 (11.3)	216 (10.7)	51 (52.0)	89 (75.4)	140 (64.8)	.000
	χ2 = 7.301;	df = 4;	*p* = .121	χ2 = 109; df = 4; *p* = < 0.001			
**Educational level**							
No education	86 (8.9)	134 (12.9)	220 (10.9)	45 (52.3)	97 (72.4)	142 (64.5)	.002
Primary school	272 (28.3)	270 (26.1)	542 (26.9)	95 (34.9)	142 (52.6)	237 (43.7)	.000
Partial high-school	272 (28.3)	46 (23.8)	518 (25.7)	75 (27.6)	84 (34.1)	159 (30.7)	.105
High-school dip^a^	211 (22.0)	256 (24.7)	467 (23.1)	35 (16.6)	69 (27.0)	104 (22.2)	.007
Academic	120 (12.5)	129 (12.5)	249 (12.3)	26 (21.7)	33 (25.6)	59 (23.7)	.468
Missing	10	12	22 (1.1)				
	χ2 = 13.722;	df = 4	*P* = .008	χ2 = 153.8; df = 4; *p* = < 0.001			
**Poverty line**							
Above	440 (52.1)	432 (48.6)	872 (43.2)	116 (26.4)	146 (33.8)	262 (30.0)	.017
Below	404 (47.9)	457 (51.4)	861 (42.7)	131 (32.4)	208 (45.5)	339 (39.4)	.000
Missing	127	158	285 (14.1)				
	χ2 = 2.169;	df = 1;	*p* = .141	χ2 = 16.6; df = 1; *p* = < 0.001			
**District**							
Northern District	515 (53.0)	545 (52.1)	1060 (52.5)	117 (22.7)	192 (36.1)	309 (29.5)	.001
Haifa District	190 (19.6)	214 (20.4)	404 (20.0)	47 (24.7)	88 (41.7)	135 (33.7)	.001
Central District	142 (14.6)	147 (14.0)	289 (14.3)	55 (38.7)	77 (52.7)	132 (45.8)	.017
Southern District	124 (12.8)	141 (13.5)	265 (13.1)	58 (46.8)	73 (52.5)	131 (49.8)	.248
	χ2 = .596	df = 3	*p* = .899	χ2 = 37.364; df = 3; *p* = < 0.001			
**BMI**							
Low/healthy	299 (31.4)	463 (46.5)	762 (37.8)	86 (28.8)	159 (34.3)	245 (32.2)	.107
Overweight	496 (52.2)	348 (35.0)	844 (41.8)	134 (27.0)	143 (41.1)	277 (32.8)	.000
Obese	156 (16.4)	184 (18.5)	340 (16.8)	51 (32.7)	108 (58.7)	159 (46.8)	.000
Missing	20	52	72 (3.6)				
	χ2 = 62.592	df = 4;	*p* = < 0.001	χ2 = 25.3; df = 2; *p* = < 0.001			
**Chronic disease**							
None	685 (71.1)	714 (68.7)	1399 (69.3)	158 (23.1)	221 (31.0)	379 (27.1)	.001
One	134 (13.9)	125 (12.0)	259 (12.8)	48 (35.8)	70 (56.0)	118 (45.6)	.001
Two or more	144 (15.0)	200 (19.2)	344 (17.0)	66 (45.8)	135 (67.5)	201 (58.4)	.000
Missing	8	8	16 (0.8)				
	χ2 = 7.155;	df = 2	*p* = .028	χ2 = 134.4; df = 2;***p*** = < 0.001			

^a^High school diploma

More than half of the study population, adults over 20 years of age, live in the Northern District, 20% in the Haifa District, 14.3% in the Central District and 13.1% in the Southern District.

Body mass index (BMI) was found to differ by gender: more women than men were in the low/healthy weight category (*p* = <.001, while more men were in the overweight category (*p* = <.001).

12.8% of our study population reported one chronic disease (13.9% of men and 12.0% of women), and 17% reported two or more chronic diseases. More women than men reported two or more chronic diseases (15.0% of men and 19.2% of women, *p* = .032). The prevalence of chronic disease was also highly associated with educational level: among those with no education (*N* = 216), 24.5% had no diseases, 16.7% had one disease and 58.8% had two or more diseases, while among those with a high school diploma (*N* = 462), 86.8% had no diseases, 9.5% had one chronic disease and 4.9% had two or more conditions (χ^2^ = 485.2; df = 8; *p* = .000) (data not shown).

### Psychological distress

Table 1 presents rates of high psychological distress by gender, according to the same socio-demographic and health-related characteristics. One of the main risk factors for psychological distress was female gender: among women 41.1% reported high psychological distress as compared to 28.5% among men. (*p* = <.001). Rates of high psychological distress increased significantly with age (*p* = <.001), in both men and women, with women having higher rates than men in all age categories, but more significantly so in the older age groups. For instance, in the 45–64 age group the rates were 47.8% among women and 29.2% among men (*p* = <.001), and in the 65+ age group the rates were 75.4% among women and 52.0% among men (*p* = <.001).

Rates of psychological distress were significantly higher among those with low levels of education than those with secondary and academic education. Among those with no education, primary school level and among those with a high school diploma, women had higher psychological distress rates than men (*p* = .002, *p* = <.001 and *p* = .007, respectively), but no significant gender differences in psychological distress were found among those with partial high school or an academic education. Women, both above and below the poverty line, reported higher psychological distress than men, but more so among those below the poverty line where 32.4% of men and 45.5% of women reported high psychological distress (*p* = <.001). Psychological distress was higher in the Southern and Central districts than in the Northern and Haifa districts. Gender differences were found in the Northern, Haifa and Central districts with women having higher rates than men, while in the Southern District the gender differences were not significant.

Psychological distress was found to be higher among those in the obese category as compared to those in the low/healthy and overweight categories (46.8% vs 32.2 and 32.8%, respectively, *p* = <.001). Women in the overweight and obese categories had significantly higher rates of high psychological distress than men (41.1% of women vs. 27.0% of men, and 58.7% of women vs. 32.7% of men, respectively, *p* = <.001). Psychological distress was found to increase with increasing number of chronic diseases in both men and women. Rates of psychological distress were higher among women than men in all categories of chronic disease: 31.0% of women without a chronic disease reported high distress, as compared to 56.0 and 67.5% among those with one or two chronic diseases, respectively. Among men, the corresponding rates were 23.1, 35.8 and 45.8%, respectively (*p* = <.001).

When included in a multivariate analysis, gender, educational level, geographical district, obesity and the presence of chronic disease remained significantly associated with high psychological distress but age did not (Table 2). Women were 1.8 times more likely than men to report high distress, and subjects with little or no education were 2.1 and 3.5 times more likely, respectively, to report distress than those with academic education. Subjects living in the Northern district were twice as likely as those living in the Haifa district to report high distress, while those living in the Central and Southern districts did not significantly differ from those in the Haifa district. Subjects classified as obese were 1.4 times more likely than normal-weight subjects to report psychological distress, and those with one or two or more chronic diseases were 2.0 and 2.1 times more likely, respectively, to report distress than those who reported no chronic diseases. In order to test whether the addition of poverty to the model would modify these findings, we performed a separate analysis that included poverty as a variable in the multivariate analysis. The addition of poverty did not significantly change the findings as reported in Table 2; however, it reduced the number of subjects by 14%. Thus, we only present the multivariate analysis, which includes all the subjects (Table 2).

**Table 2:** High psychological distress among Palestinian citizens of Israel by gender, age, educational level, BMI, chronic disease and geographical district. Logistic regression analysis. (OR, 95% CI)

Risk factors	High psychological distress^a^		
	OR	(95%CI)	*p*
*Gender*			
Male	1.00 [reference]		
Female	1.78	(1.4–2.2)	<.001
*Age group*			
18–24	1.00 [reference]		
25–34	0.96	(0.7–1.3).	.811
35–44	0.75	(0.5–1.1)	.122
45–64	0.64	(0.4–1.0)	.027
65≥	1.23	(0.7–2.1)	.447
*Educational level*			
No education	3.50	(2.1–5.6)	<.001
Primary school	2.09	(1.4–3.0)	<.001
Partial high school	1.39	(1.0–2.0)	.071
High school diploma	0.81	(0.6–1.2)	.334
Academic	1.0 [reference]		
*Geographical district*			
Northern district	1.0 [reference]		
Haifa district	0.55	(0.4–0.7)	<.001
Central district	1.10	(0.8–1.5)	.523
Southern district	0.88	(0.6–1.2)	.436
*BMI*			
Low/healthy	1.00 [reference]		
Overweight	1.07	(0.8–1.4)	.574
Obese	1.39	(1.0–1.9)	.034
*Chronic disease*			
None	1.00 [reference]		
One	1.92	(1.4–2.6)	<.001
Two or more	2.09	(1.5–3.0)	<.001

^a^global score of ≥17

Figure 1 presents a comparison between GHQ-12 scores obtained by the HESPI with those obtained by Ponizovsky et al., (2018) who analyzed INHS data gathered in 2003–2004. The INHS used the 0,1,2,3 rating for the GHQ-12 items and obtained a total GHQ-12 score that ranged from 0 to 36. Within this range, the authors state that that scores of “11 -12 are typical, a score of over 15 – 19 suggests moderate distress and a score of 20 or more suggests severe problems and psychological distress” [[18], p. 727]. They divided their population into four groups and assigned them the following categories, according to their global score: asymptomatic (score 0–10), sub-clinically symptomatic (score 11–14), symptomatic (score 15–19) and highly symptomatic (score 20–36). For comparison purposes, we converted the GHQ-12 global scores of our subjects into the same categories used by the INHS.

The main finding is that the HESPI and the INHS populations differ significantly with respect to rates of psychological distress (χ^2^ = 1207.9; *p* = ≤0.001). The HESPI study reported significantly higher prevalence rates in the ‘symptomatic’ and ‘highly symptomatic’ GHQ-12 categories than the INHS (22.2 and 22.9% vs 7.5 and 6.5%, respectively).

### Self-rated health

Table 3 presents rates of self-rated health by socio-demographic and health-related risk factors. In general, 36.4% of adults rated their health as ‘excellent’, 35.8% as ‘good’, 15.6% as ‘not so good’ and 12.2% as ‘poor’. No gender differences were found (*p* = .108). A direct association was seen with age: as age increased, the percentage of those rating their health as ‘poor’ or ‘not so good’ also increased. For both male and female adults, the percentage that assessed their health as ‘excellent’ decreased significantly with age. Among those 65 years of age or more, only 22.5% of males and 16.1% of females assess their health as ‘good’ or ‘excellent’, while 34.7% of males and 50.8% of females considered it to be ‘poor’ (*p* = <.001).

**Table 3:** Self-rated health among Palestinian citizens of Israel by socio-demographic and health-related factors

Self-rated health																
Socio-demographic and health-related factors				Poor			Not so good			Good			Excellent			
				N%			N %			N %			N %			
				246 (12.2)			315 (15.6)			723 (35.8)			734 (36.4)			
Male	Female	Total		M	F	x̅	M	F	x̅	M	F	x̅	M	F	x̅	*p* value*
n 971	1047	2018		103 (10.6)	143 (13.7)		150 (15.4)	165 (15.8)		345 (35.5)	378 (36.1)		373 (38.4)	361 (34.4)		
				χ2 = 6.067; df = 3; *p* = < 0.108												
**Age**																
18–24		137	167	.7	3.0	2.0	5.8	5.4	5.6	26.4	26.9	26.6	67.2	64.7	65.8	.559
25–34		237	285	4.6	3.9	4.2	7.6	4.2	5.7	33.3	37.9	35.8	54.4	54.0	54.2	.318
35–44		249	228	6.0	10.1	8.0	6.4	10.1	8.2	49.4	52.2	50.7	38.2	27.6	33.1	.035
45–64		250	249	16.8	17.7	17.2	26.4	32.9	29.7	35.2	36.9	36.1	21.6	12.4	17.0	.044
65≥		98	118	34.7	50.8	43.5	42.9	33.1	37.5	19.4	11.9	15.3	3.1	4.2	3.7	.080
				χ2 = 739.225; df = 12; *p* = < 0.001												
**Educational level**																
No education		86	134	30.2	41.8	37.3	37.2	30.6	33.2	19.8	20.9	20.5	12.8	6.7	9.1	.193
Primary school		272	270	15.8	18.5	17.2	23.5	25.9	24.7	35.3	36.3	35.8	25.4	19.3	22.3	.362
Partial high-school		272	246	7.7	8.5	8.1	9.9	10.6	10.2	41.9	45.1	43.3	40.4	35.8	38.2	.753
High-school diploma		211	256	3.3	4.7	4.1	8.1	7.8	7.9	30.3	35.2	33.0	58.3	52.3	55.0	.551
Academic		120	129	4.2	2.3	3.2	8.3	5.4	6.8	41.7	36.4	39.0	45.8	55.8	51.0	.380
				χ2 = 443.336; df = 12; *p* = < 0.001												
**Poverty**																
Above poverty line		440	432	9.5	6.5	8.0	12.5	14.4	13.4	35.2	40.7	38.0	42.7	38.4	40.6	.119
Below poverty line		404	457	10.2	17.9	14.6	16.3	14.4	15.3	35.9	35.0	35.4	36.9	32.6	34.6	.030
				χ2 = 22.707; df = 3; *p* = < 0.001												
**Geographical district**																
Northern district		517	543	10.4	14.2	12.4	17.0	15.5	16.2	37.5	35.5	36.5	35.0	34.8	34.9	.299
Haifa district		205	199	11.2	12.1	11.6	12.7	19.6	16.1	35.6	33.7	34.7	40.5	34.7	37.6	.259
Central district		124	165	8.9	15.8	12.8	9.7	8.5	9.0	28.2	38.2	33.9	53.2	37.6	44.3	.033
Southern district		125	140	12.0	11.4	11.7	19.2	20.0	19.6	34.4	39.3	37.0	34.4	29.3	31.7	.798
				χ2 = 18.203; df = 9; *p* = 0.033												
**BMI**																
Low/healthy		229	463	12.0	9.1	10.2	12.7	8.9	10.4	29.4	33.9	32.2	45.8	48.2	47.2	.138
Overweight		496	348	9.3	10.9	10.0	15.5	16.4	15.9	36.3	43.1	39.1	38.9	29.6	35.1	.043
Obese		156	184	9.6	28.3	19.7	17.9	28.3	23.5	46.2	30.4	37.6	26.3	13.0	19.1	<.001
				χ2 = 107; df = 6; < 0.001												
**Chronic disease**																
None		685	714	2.3	2.8	2.6	5.0	4.3	4.6	40.1	44.8	42.5	52.6	48.0	50.3	.284
One		134	125	20.1	22.4	21.2	42.5	38.4	40.5	29.9	27.2	28.6	7.5	12.0	9.7	.580
Two or more		144	200	41.0	46.0	43.9	39.6	42.0	41.0	17.4	10.5	13.4	2.1	1.5	1.7	.294
				χ2 = 1071.481; df = 6; *p* = < 0.001												
**Psychological distress**																
Low distress		694	617	5.8	5.0	5.4	13.3	12.2	12.7	36.6	40.2	38.3	44.4	42.6	43.6	.578
High distress		277	430	22.7	26.0	24.8	20.9	20.9	20.9	32.9	30.2	12.3	23.5	22.8	23.1	.766
				χ2 = 220.049; df = 3; *p* = < 0.001												

*for gender differences within each sub-category

A reverse pattern was observed with educational level: as educational level increased, the percentage that rated their health as ‘poor’ or ‘not so good’ decreased, whereas the percentage who rated their health as excellent increased (*p* = <.001). For those above the poverty line 21.4% rated their health as ‘poor’ or ‘not so good’, whereas for those below the poverty line the rate was 29.9% (*p* = <.001).

No clear pattern of SRH could be identified by geographical district. More men than women in all the districts rated their health as ‘excellent’. Among those classified as ‘obese”, 19.7% rated their health as “poor”, as compared with 10.0 and 10.2% in the “overweight” and “healthy” BMI categories, respectively (*p* = <.001).

A strong association was found between number of chronic diseases and self-rated health: among those with no chronic diseases, 2.6% rated their health as ‘poor’ and 50.3% as ‘excellent’, while among those with one disease 21.2% rated their health as ‘poor’ and 9.7% as ‘excellent’ and among those with two or more chronic diseases 43.9% rated their health as ‘poor’ and 1.7% as ‘excellent’ (*p* = <.001).

Psychological distress was also associated with SRH. Among those with low distress, 5.4% rated their health as ‘poor’ and 43.6% as ‘excellent’. Among those with high distress, 24.8% rated their health as ‘poor’ and 23.1% as ‘excellent’ (*p* = <.001). For most of these variables, gender differences in SRH were not significant, except for those in the 35–64 age groups, those below the poverty line and those in the overweight and obese categories. In all of these, rates of poor SRH were higher for women than for men.

Table 4 shows that for Palestinian citizens of Israel, belonging to the older age groups, having a low educational level, having one or more chronic diseases, as well as reporting high psychological distress, were independent risk factors for rating their health as ‘poor’, also when including the other variables in the regression analysis. Obesity, which was found to be significantly associated with self-rated health in the bivariate analyses, was not associated with ‘poor’ self-rated health when including the other variables in the multivariate analysis (*p* = .605). With respect to age, subjects between 45 and 64 years of age or 65 or more were 2.4 and 3.6 times more likely, respectively, to rate their health as ‘poor’ than those in the 18–24 age group (*p* = .005 and *p* = .001, respectively). Those with primary school level were 2.1 times as likely to rate their health as ‘poor’ than those with an academic education (*p* = .010). Subjects reporting high psychological distress were 2.5 times more likely than those with low distress to rate their health as ‘poor’ (*p* = <.001). The strongest risk factor for self-rating health as ‘poor’ was the presence of one or more chronic diseases: among those subjects with one chronic disease the risk was 13.2 times more likely than among those with no disease and among those with two chronic diseases or more the risk was 29.7 times higher (*p* = <.001 and *p* = <.001, respectively).

**Table 4:** Poor’ self-rated health among Palestinian citizens of Israel, by age, educational level, geographical district, BMI, chronic disease and GHQ score. Logistic regression analysis. (OR, 95% CI)

Risk factors	‘Poor’ self-rated health		
	OR	(95%CI)	p
*Age group*			
18–24	1.00 [reference]		
25–34	1.23	(0.7–2.2)	.495
35–44	1.29	(0.7–2.4)	.394
45–64	2.40	(1.3–4.4)	.005
65≥	3.56	(1.6–7.8)	.001
*Educational level*			
No education	2.02	(1.0–4.2)	.055
Primary school	2.14	(1.2–3.8)	.009
Partial high school	1.77	(0.9–3.2)	.052
High school diploma	1.48	(0.8–2.7)	.208
Academic	1.0 [reference]		
*Geographical district*			
Northern District	1.0 [reference]		
Haifa District	1.39	(0.9–2.0)	.102
Central District	0.70	(0.4–1.1)	.149
Southern District	1.15	(0.7–1.8)	.525
*BMI*			
Low/healthy	1.00 [reference]		
Overweight	0.84	(0.6–1.2)	.323
Obese	0.90	(0.6–1.4)	.632
*Chronic disease*			
None	1.00 [reference]		
One	13.59	(9.4–19.6)	<.001
Two or more	30.21	(19.6–46.6)	<.001
*Psychological distress*			
Low	1.00 [reference]		
High	2.55	(1.9–3.5)	<.001

## Discussion

This study, which addresses the broad complexity of factors associated with psychological distress and poor self-assessment of health, has given us the opportunity to identify some of the most salient risk factors for these constructs in the Palestinian minority in Israel, using the GHQ-12 and SRH measures, respectively. Although the risk factors for psychological distress and poor SRH somewhat overlap, the extent of the risk associated with each of the factors is very different for the two constructs. For psychological distress, sociodemographic factors have a greater impact, while for poor SRH the existence of actual chronic conditions is the strongest predictor.

With respect to psychological distress, as measured by the GHQ-12 scale, mean global scores in our population were 20.7 for women and 17.5 for men. Multivariate analysis showed that the risk of high psychological distress was greater among women, those with low education, those in the obese weight category and those with one or more chronic health conditions, over and above the effect of the other variables.

Our finding that high psychological distress in our study was strongly associated with low education (subjects with no education at all or only primary school level were 3.4 and 2.1 times more likely, respectively, to report high psychological distress than subjects with academic education), is in accordance with other studies conducted in a variety of populations [9, 21], and among the Palestinians in Israel in particular [24]. For the Palestinian population of Israel, this finding is one with a high potential impact, since in this minority population group, 38% were categorized as having a very low educational level (11% reported having no formal education at all, and a further 27% reported having only a primary school educational level). This, in comparison with 12.3% of the Jewish Israeli population [57].

Although poverty, defined in or study as being below the poverty line according to income, was strongly associated with psychological distress in the univariate analysis, it is not presented in our multivariate analysis due to a high percentage of missing values (14.1%). It should be pointed out, however, that almost 50% of the Arab population in Israel are below the poverty line, as compared with 27.8% in the general population [29], putting a large proportion of the population at risk of psychological distress.

With respect to gender, our findings are similar to those shown by other studies, in which women are consistently reported to have higher rates of psychological distress [15, 16].. It is also known that the impact of poverty and financial stress is particularly evident among women. This has been attributed to higher sensitivity of women than men to the effects of poverty, poor neighborhood or city conditions, financial stress and level of debt, which increase the risk of depression [58]. Alternatively, higher psychological distress has been attributed to a response bias, that is, the possibility that women express their distress “more freely than men” [14, 59].

As expected, based on results of previous studies [24], psychological distress rates were higher among the Palestinian citizens of Israel than among the general Israeli population. In comparing rates of psychological distress among Palestinian citizens of Israel in our study with those of the general Israeli population (results presented by Ponizovsky et al., 2018), we indeed found that the Palestinians in the HESPI population presented a very different distribution from that emerging from the INHS data reflecting the general Israeli population [18]. The most striking difference was that 75% of the general population in the INHS study were classified as “asymptomatic” (as compared with 30% classified as asymptomatic in the Palestinian minority) and the proportion of symptomatic and highly symptomatic was 14% in the general Israeli population (as compared with 45% in the Palestinian population).

The Palestinian minority population in many respects can be seen as a distinct group that does not share many of the characteristics of the general Israeli population. A comparison of the sociodemographic traits of the Palestinian minority in this study and the general Israeli population shows that many of the known risk factors for psychological distress are more prevalent among the Palestinian citizens of Israel, including low educational level [11, 18, 36], higher rates of poverty [29] and higher rates of chronic disease [33]; and this may well explain in part the discrepancies in rates of psychological distress.

The overall profile emerging from this study is one of poverty, which affects women more than men, and of a population with a relatively low educational level and high rates of chronic morbidity. Phelan et al. (2010) propose that resources such as money, knowledge, prestige, power and beneficial social connections are relevant to protect health in any population, and these resources are largely missing among Palestinians in Israel [60]. This composite picture of a disadvantaged minority population is in accordance with the approach of Veenstra (2011) [26], and Dogra, 2012 [61], who emphasized the importance of considering the complex interplay between multiple stressors, which are mutually reinforcing one another, being, as they are, intrinsically entwined.

With respect to self-rated health, we found that 12.2% of the Palestinians in Israel rated their health as “poor”. The strongest risk factor for “poor” SRH in our study population was the presence of one or two or more chronic health conditions (OR = 13.2 and OR = 29.7, respectively). To a lesser degree, older age, low educational level and high psychological distress also remained significantly associated with ‘poor’ self-rated health over and above the effect of the other variables.

We found that the risk factors associated with poor self-rated health were different from those associated with high psychological distress; these two constructs, although strongly associated (i.e., among those with high distress, 24.8% rated their health as ‘poor’ as compared with 5.4% among those with low distress), were differently impacted by the risk factors included in our study. While the strongest predictors for high psychological distress were found to be related to socio-demographic characteristics, i.e., low education and female gender, as noted above, the strongest predictor for self-rating health as ‘poor’ was actual health status, i.e., the presence of one or more chronic diseases. Our findings present a portrait of an ethnic minority in which those with chronic diseases are many times more likely to rate their health as ‘poor’ than those with no chronic diseases. In our population, the presence of chronic conditions was strongly associated with lower education (36.9% of those with no education as compared to 2.9% among those with academic education). Chronic conditions in Israel have also been strongly associated with older age, female gender, a monthly household income of NIS 3000 or less, and overweight [33].

The presence of chronic conditions may be seen as an indicator that sums up the complex picture of a disadvantaged minority group and of the multiple factors and stressors that contribute to the poor self-assessment of health. In any case, this strong association between ‘poor’ SRH and number of chronic conditions seems to clarify to some degree the question posed by Bailis, Segall & Chipperfield (2003) of what it is that SRH measures: whether it reflects one’s health status or whether it is the expression of an enduring self-concept [47]. Our study provides strong evidence that in the Palestinian population SRH indeed reflects one’s health status, as evidenced by the increased risk of rating one’s health as ‘poor’ when chronic disease is present and the dose-response nature of this association.

### Limitations of the study

In the comparison between our findings and those of the INHS, it must be pointed out that the INHS was carried out in 2003–2004 and therefore more than 10 years before the HESPI. This time difference may also explain the higher psychological distress in our more recent study, perhaps reflecting a secular trend, including increasing distress in recent times due to socio-cultural, political and economic changes. Another limitation of our study is the relatively high percentage of cases with missing data regarding income and poverty level (14.1% of subjects; *n* = 285). This compromised to some extent the multivariate analyses for both psychological distress and self-rated health, since these analyses did not include poverty. On the other hand, low educational level (which was included in the multivariate analyses), is strongly associated with poverty and to some extent can serve as a proxy measure for this variable.

## Conclusions and recommendations

The characteristics of the study population, which suffers from multiple stressors related to socio-economic disadvantage, adverse living circumstances and limited access to mental health care, require that we pay particular attention to the statement of Dogra et al. (2012), that there is “a complex interplay between minority status and social class, with terms such as ethnicity being a proxy for multifaceted sociocultural and economic variables” [[60], p. 265].

. It is evident that the burden of chronic disease in this population, which has a higher percentage of uneducated and poor citizens, is disproportionate and should be addressed by the relevant state institutions. Concrete intervention plans need to be prepared by the Ministry of Education, with the aim of improving the educational status of future generations, the basic condition for increasing their opportunities for achieving an enhanced economic status and better physical and mental health. As well, both the Ministry of Education and the Ministry of Health should join efforts to carry out health education and health promotion interventions for school children and adolescents in order to improve their health literacy today and in the future. Special attention should be paid to the population in the Southern District, where the most disadvantaged Arab citizens live. The needs of the Bedouin ‘unrecognized villages’, which lack infrastructure and accessible education and health services, should be met and their civil rights recognized.

Clearly, the associations between minority status, deprivation and poverty on the one hand and psychological distress and poor self-rated health on the other, are not unique to the Palestinian citizens of Israel and therefore this study will allow for the examination and generalization of the current findings to other discriminated and disadvantaged minority populations.

## Supplementary Information

**Additional file 1:.** Results section.

## Data Availability

All data generated or analyzed during this study are included in this published article and its supplementary information files.

## Abbreviations

GHQ-12: General health questionnaire-12
SRH: Self-rating of health
DSM-IV: Diagnostic and statistical manual of mental disorders, 4th edition
ICD-10: International classification of diseases, 10th revision
INHS: Israel national health survey
INHIS: Israeli national health interview survey
HESPI: Health and environment survey of palestinian citizens of Israel
CBS: Central bureau of statistics
PSU: Preliminary sampling units
BMI: Body mass index
NII: National insurance institute
