# Supplementary material for: Correlates of psychological distress and self-rated health among Palestinian citizens of Israel: findings from the health and environment survey (HESPI)
Source: Isr J Health Policy Res. 2021 Jan 20;10:3. doi: 10.1186/s13584-021-00439-z (PMC7818903; doi:10.1186/s13584-021-00439-z)
Supplement: Supplementary file 1 — Additional file 1:. Results section. [file 13584_2021_439_MOESM1_ESM.docx]

**Results section**

*Characteristics of the population:* Table 1, in the left side columns, shows the distribution of selected characteristics of the population by gender. 15.1% of the sample were between 18 and 24 years of age and 10.7% were aged 65 or above. No gender differences in age were found. Educational level differed significantly by gender: more women than men had no education at all (p=.011), while more men than women had only partial high school studies (p= <.001). There were no gender differences regarding achievement of a high school diploma or an academic education (12.5% of the study population). Educational level also differed by age, since 19.5% of those aged 25 -44 had an academic education compared to 1.4% of those aged 65 or more and only 1.4% of those aged 25 – 44 had no education as compared to 59.5% of those aged 65 or more (χ^2^  =1042.6; df= 16; p =< .001) (data not shown). A striking trait of this population is the high level of poverty: 42.7% of the study population were defined as being below the poverty line; no gender differences were found. It must be stressed here that poverty data, based on income information, is missing for 14.1% of the subjects (127 men and 158 women).

More than half of the study population, adults over 20 years of age, live in the Northern District, 20% in the Haifa District, 14.3% in the Central District and 13.1% in the Southern District.

Body mass index (BMI) was found to differ by gender: more women than men were in the low/healthy weight category (p=<.001, while more men were in the overweight category (p= <.001).

12.8% of our study population reported one chronic disease (13.9% of men and 12.0% of women), and 17 % reported two or more chronic diseases. More women than men reported two or more chronic diseases (15.0% of men and 19.2% of women, p=.032). The prevalence of chronic disease was also highly associated with educational level: among those with no education (N=216), 24.5% had no diseases, 16.7% had one disease and 58.8% had two or more diseases, while among those with a high school diploma (N=462), 86.8% had no diseases, 9.5% had one chronic disease and 4.9% had two or more conditions (χ^2^ = 485.2; df=8; p=.000) (data not shown).

*Psychological distress.*

Table 1 presents rates of high psychological distress by gender, according to the same socio-demographic and health-related characteristics. One of the main risk factors for psychological distress was female gender: among women 41.1% reported high psychological distress as compared to 28.5% among men. (p= <.001). Rates of high psychological distress increased significantly with age (p= <.001), in both men and women, with women having higher rates than men in all age categories, but more significantly so in the older age groups. For instance, in the 45-64 age group the rates were 47.8% among women and 29.2% among men (p= <.001), and in the 65+ age group the rates were 75.4% among women and 52.0% among men (p=<.001).

Rates of psychological distress were significantly higher among those with low levels of education than those with secondary and academic education. Among those with no education, primary school level and among those with a high school diploma, women had higher psychological distress rates than men (p = .002, p=<.001 and p=.007, respectively), but no significant gender differences in psychological distress were found among those with partial high school or an academic education. Women, both above and below the poverty line, reported higher psychological distress than men, but more so among those below the poverty line where 32.4% of men and 45.5% of women reported high psychological distress (p=<.001). Psychological distress was higher in the Southern and Central districts than in the Northern and Haifa districts. Gender differences were found in the Northern, Haifa and Central districts with women having higher rates than men, while in the Southern District the gender differences were not significant.

Psychological distress was found to be higher among those in the obese category as compared to those in the low/healthy and overweight categories (46.8% vs 32.2% and 32.8%, respectively, p=<.001). Women in the overweight and obese categories had significantly higher rates of high psychological distress than men (41.1% of women vs. 27.0% of men, and 58.7% of women vs. 32.7% of men, respectively, p=<.001). Psychological distress was found to increase with increasing number of chronic diseases in both men and women. Rates of psychological distress were higher among women than men in all categories of chronic disease: 31.0% of women without a chronic disease reported high distress, as compared to 56.0% and 67.5% among those with one or two chronic diseases, respectively. Among men, the corresponding rates were 23.1%, 35.8% and 45.8%, respectively (p= <.001).

**(Table 1 about here)**

When included in a multivariate analysis, gender, educational level, geographical district, obesity and the presence of chronic disease remained significantly associated with high psychological distress but age did not (Table 2). Women were 1.8 times more likely than men to report high distress, and subjects with little or no education were 2.1 and 3.5 times more likely, respectively, to report distress than those with academic education. Subjects living in the Northern district were twice as likely as those living in the Haifa district to report high distress, while those living in the Central and Southern districts did not significantly differ from those in the Haifa district. Subjects classified as obese were 1.4 times more likely than normal-weight subjects to report psychological distress, and those with one or two or more chronic diseases were 2.0 and 2.1 times more likely, respectively, to report distress than those who reported no chronic diseases. In order to test whether the addition of poverty to the model would modify these findings, we performed a separate analysis that included poverty as a variable in the multivariate analysis. The addition of poverty did not significantly change the findings as reported in table 2; however, it reduced the number of subjects by 14%. Thus, we only present the multivariate analysis, which includes all the subjects (Table 2).

**(Table 2 about here)**

Figure 1 presents a comparison between GHQ-12 scores obtained by the HESPI with those obtained by Ponizovsky et al., (2018) who analyzed INHS data gathered in 2003 - 2004. The INHS used the 0,1,2,3 rating for the GHQ-12 items and obtained a total GHQ-12 score that ranged from 0 to 36. Within this range, the authors state that that scores of "11 -12 are typical, a score of over 15 – 19 suggests moderate distress and a score of 20 or more suggests severe problems and psychological distress" [18, p. 727]. They divided their population into four groups and assigned them the following categories, according to their global score: asymptomatic (score 0 -10), sub-clinically symptomatic (score 11 -14), symptomatic (score 15 – 19) and highly symptomatic (score 20 -36). For comparison purposes, we converted the GHQ-12 global scores of our subjects into the same categories used by the INHS.

The main finding is that the HESPI and the INHS populations differ significantly with respect to rates of psychological distress (χ^2^= 1207.9; p = ≤0.001). The HESPI study reported significantly higher prevalence rates in the 'symptomatic' and 'highly symptomatic' GHQ-12 categories than the INHS (22.2% and 22.9% vs 7.5% and 6.5%, respectively).

**(Figure 1 about here)**

*Self-rated health*

Table 3 presents rates of self-rated health by socio-demographic and health-related risk factors. In general, 36.4% of adults rated their health as 'excellent', 35.8% as 'good', 15.6% as 'not so good' and 12.2% as 'poor'. No gender differences were found (p=.108). A direct association was seen with age: as age increased, the percentage of those rating their health as 'poor' or 'not so good' also increased. For both male and female adults, the percentage that assessed their health as 'excellent' decreased significantly with age. Among those 65 years of age or more, only 22.5% of males and 16.1% of females assess their health as 'good' or 'excellent', while 34.7% of males and 50.8% of females considered it to be 'poor' (p=<.001).

A reverse pattern was observed with educational level: as educational level increased, the percentage that rated their health as 'poor' or 'not so good' decreased, whereas the percentage who rated their health as excellent increased (p=<.001). For those above the poverty line 21.4% rated their health as 'poor' or 'not so good', whereas for those below the poverty line the rate was 29.9% (p=<.001).

No clear pattern of SRH could be identified by geographical district. More men than women in all the districts rated their health as 'excellent'. Among those classified as 'obese”, 19.7% rated their health as “poor”, as compared with 10.0% and 10.2% in the “overweight” and “healthy” BMI categories, respectively (p=<.001).

A strong association was found between number of chronic diseases and self-rated health: among those with no chronic diseases, 2.6% rated their health as 'poor' and 50.3% as 'excellent', while among those with one disease 21.2% rated their health as 'poor' and 9.7% as 'excellent' and among those with two or more chronic diseases 43.9% rated their health as 'poor' and 1.7% as 'excellent' (p=<.001).

Psychological distress was also associated with SRH. Among those with low distress, 5.4% rated their health as 'poor' and 43.6% as 'excellent'. Among those with high distress, 24.8% rated their health as 'poor' and 23.1% as 'excellent' (p=<.001). For most of these variables, gender differences in SRH were not significant, except for those in the 35 -64 age groups, those below the poverty line and those in the overweight and obese categories. In all of these, rates of poor SRH were higher for women than for men.

**(Table 3 about here)**

Table 4 shows that for Palestinian citizens of Israel, belonging to the older age groups, having a low educational level, having one or more chronic diseases, as well as reporting high psychological distress, were independent risk factors for rating their health as 'poor', also when including the other variables in the regression analysis. Obesity, which was found to be significantly associated with self-rated health in the bivariate analyses, was not associated with 'poor' self-rated health when including the other variables in the multivariate analysis (p=.605). With respect to age, subjects between 45 and 64 years of age or 65 or more were 2.4 and 3.6 times more likely, respectively, to rate their health as 'poor' than those in the 18 – 24 age group (p=.005 and p=.001, respectively). Those with primary school level were 2.1 times as likely to rate their health as 'poor' than those with an academic education (p=.010). Subjects reporting high psychological distress were 2.5 times more likely than those with low distress to rate their health as 'poor' (p=<.001). The strongest risk factor for self-rating health as 'poor' was the presence of one or more chronic diseases: among those subjects with one chronic disease the risk was 13.2 times more likely than among those with no disease and among those with two chronic diseases or more the risk was 29.7 times higher (p=<.001 and p=<.001, respectively).

**(Table 4 about here)**
